# Vaping and smoking as risk factors for arthritis: Evidence from double machine learning

**DOI:** 10.1371/journal.pone.0360863

**Published:** 2026-10-23

**Authors:** Onyedikachi Emmanuel Chike, Syed Badruddoza, Conrad Lyford

**Affiliations:** 1 The Rinker College of Business, Palm Beach Atlantic University, West Palm Beach, Florida, United States of America; 2 Department of Agricultural and Applied Economics, Texas Tech University, Lubbock, Texas, Lubbock, United States of America; University of Toronto, CANADA

## Abstract

Smoking is a well-documented risk factor for chronic diseases, including arthritis. Vaping, often marketed as a safer alternative, has risen sharply, yet its implications for musculoskeletal health remain unclear. We examine the associations of vaping and smoking with arthritis using Double Machine Learning (DML), and present illustrative economic scenarios projecting the potential cost implications of arthritis cases associated with these behaviors. Data from the Behavioral Risk Factor Surveillance System (BRFSS) of the Centers for Disease Control and Prevention (CDC), collected in the United States, for adults aged 18–64 years (N = 36,768) is used. Arthritis is the outcome variable; vaping and smoking are the exposure variables, with estimates conditioned on twenty-seven demographic, behavioral, and health-related covariates (See Table 1). Results show that vaping is associated with a 2.12 percentage point higher prevalence of arthritis than among non-vapers, while smoking is associated with a 2.22 percentage points increase compared with non-smokers. When accounting for concurrent nicotine behaviors, the estimated associations remain largely stable, with smoking exhibiting a slightly larger estimate of 2.14 percentage points while vaping attenuated to 1.91 percentage points. Vaping was estimated to be associated with approximately 190,581–254,109 arthritis cases annually, corresponding to a potential economic cost projection of $133.4--$177.9 million. Smoking was statistically associated with approximately 518,886–687,413 cases, corresponding to $363.2 –$481.2 million. Combined, the projected economic burden associated with vaping and smoking was approximately $496.6–$659.1 million annually under conservative assumptions. Relative to published estimates of the total U.S. arthritis burden, these projections represent approximately 0.09%–0.22% of arthritis-related expenditures under direct healthcare cost assumptions and up to 9.23% when broader indirect and societal costs are considered. Our findings suggest that vaping is associated with musculoskeletal risk comparable to, or potentially exceeding, that of smoking, and do not support the perception of vaping as a risk-free alternative. The illustrative projections may inform policy discussions on vaping and smoking in relation to the broader arthritis-related health and economic burden.

## Introduction

Smoking remains a major public health concern, with well-documented psychological and physical harms and strong links to cardiovascular disease, lung cancer, respiratory conditions, and other chronic illnesses [1–10]. Although decades of tobacco-control policies have reduced use, cigarette smoking persists: in 2021, 11.5% of U.S. adults reported current cigarette smoking [11], and more recent estimates in 2023 suggest only modest additional declines [12,13].

At the same time, the use of e-cigarettes (“Vaping”) has become increasingly common. Vaping involves inhaling an aerosol that typically contains nicotine, flavorings, and other chemicals. While many users perceive vaping as less harmful than combustible cigarettes, it still delivers additive nicotine and may expose users to potentially toxic substances, and the long-term health consequences are not yet well established. In 2021, 4.5% of U.S. adults used vaping products, and prevalence was substantially higher among young adults ages 18–24 (about 11%) [14]. Importantly, vaping is often discussed as a close substitute for smoking [15,16]. Given ongoing uncertainty about vaping’s health effects and the persistent problem of smoking-related disease, agencies such as the Centers for Disease Control and Prevention (CDC) and the National Health Service (NHS) have discussed vaping as a potential substitute for conventional cigarettes in harm-reduction efforts [17,18].

Despite the rising prevalence of vaping and cigarette smoking and growing concern about their long-term health consequences, existing evidence has focused primarily on respiratory and cardiovascular outcomes. As a result, a key research gap remains about whether, and to what extent, these tobacco-related behaviors are associated with chronic musculoskeletal conditions such as arthritis, a leading cause of pain and disability worldwide [19,20]. Several studies suggest that smoking may worsen arthritis by increasing disease activity, reducing medication effectiveness, and elevating the risk of severe complications [21–23]. However, much less is known about the association between vaping and arthritis risk.

This gap is particularly consequential given that arthritis affects more than 20% of U.S. adults and imposes substantial public health and economic problem [20]. Moreover, the limited studies linking vaping to musculoskeletal outcomes are sparse and inconsistent, reporting mixed findings ranging from potential harm to negligible effects [24–27]. Finally, few studies have illustrated the potential cost projection of arthritis associated to smoking and vaping, further limiting policymakers’ and clinicians’ ability to assess the broader societal costs of tobacco-related exposures [28].

The purpose of this study is to examine the associations between smoking, vaping, and arthritis, and to evaluate whether vaping is truly safer than smoking using data-driven evidence. In addition, we illustrate economic scenarios projecting the potential cost associated with arthritis and these behaviors for both individuals and the broader economy. Using nationally representative data from the CDC’s Behavioral Risk Factor Surveillance System (BRFSS), we apply Double Machine Learning (DML) to assess the independent and joint associations of vaping and smoking on arthritis. We also examine how each behavior influences the other’s association with arthritis. Furthermore, SHapley Additive exPlanations (SHAP) values are used to identify the marginal contributions of key predictors.

Previous studies have shown an interest in examining the relationship between vaping behavior and various health problems [27,29–42]. However, scientists and scholars have yet to agree on the possible effects of vaping relative to smoking. Proponents assert that vaping has specific benefits, including decreased exposure to far fewer toxins at lower levels than smoking cigarettes [17,18]. While vaping may seem to serve as a harm reduction strategy for current smokers, there are legitimate concerns over its appeal to non-smokers, especially young ones, and the potential normalization of nicotine consumption [43,44]. Nicotine is not without health complications. It is an immensely addictive chemical, with a level of addictiveness that can be likened to heroin, cocaine, or alcohol [45,46]. Hence, while vaping may alleviate some health hazards linked to smoking, it also presents health problems related to nicotine and other elements [47]. In addition, scholars have also explored the possible links to various kinds of arthritis, including smoking and arthritis [22,48–56], as well as vaping and arthritis, including bone-related disease [29,57–59]. The conclusions of these studies are varied.

Given the well-established adverse health effects of smoking and the ongoing debate about vaping as a potential substitute for conventional cigarette use [9,39,44], randomized controlled trials (RCTs) directly comparing these behaviors are neither feasible nor ethically justifiable. The methodological approach used in this study flexibly controls for high-dimensional confounders while preserving valid interpretation, enabling robust estimation of the independent and joint associations of smoking and vaping with arthritis, as well as an economic scenarios projecting the potential cost associations, without relying on restrictive parametric assumptions. Overall, this study contributes to the growing literature at the intersection of health economics, behavioral risk analysis, and ML–based analysis by offering robust, data-driven evidence on the health and economic consequences of smoking and vaping.

## Data

We utilize the Behavioral Risk Factor Surveillance System (BRFSS) 2016–2022 [60]. The BRFSS is a telephone survey conducted by state health departments in partnership with the CDC to gather data on overall health and behaviors related to health. This survey is done monthly using a random-digit dialing method. It draws a representative sample of adults aged 18 years and older from the civilian noninstitutionalized population, that is, individuals residing in community settings such as private households and excluding those living in institutional group quarters (e.g., nursing homes, prisons, and long-term care facilities) and collects data on a wide range of key health outcomes.

The data are collected at the individual level across all 50 states and the District of Columbia, providing a strong representation of the U.S. population. The BRFSS covers a broad geographic scope and demographic diversity, ensuring consistency with the overall U.S. population. The pooled BRFSS 2016–2022 dataset initially contained over 500,000 respondents. After restricting the sample to adults aged 18–64 years and excluding observations with missing information on the outcome, exposures, or covariates, the final complete-case analytic sample consisted of 36,768 respondents. For this study, the analytical sample is restricted to adults aged 18–64 years to focus on the working-age, noninstitutionalized population in which vaping and smoking behaviors are more prevalent and exhibit sufficient variation for reliable estimation. Among adults aged 65 and older, e-cigarette use is relatively rare in our data, leading to sparse observations and potentially unstable estimates of the joint effects of vaping and smoking. Moreover, arthritis prevalence rises sharply with age due to degenerative and comorbidity-related processes, which may introduce substantial age-driven heterogeneity and confounding that could mask behavioral associations of interest. In addition, older adults are more likely to differ systematically in healthcare access, medication use, retirement status, and survivorship patterns, all of which may be correlated with both tobacco-related behaviors and arthritis outcomes. Restricting the sample to ages 18–64, therefore, enhances internal validity, improves comparability across exposure groups, and allows for more credible estimation of the independent and joint associations between vaping, smoking, and arthritis within a behaviorally relevant population.

### Variable definition

The model for this study includes one outcome variable and two exposure variables. The outcome variable is arthritis, and the exposure variables are vaping and smoking. Table 1 presents summary statistics for the BRFSS 2016–2022 analytic sample (N = 36,768). The outcome of interest, arthritis, is reported by 36.42% of the sample, indicating a substantial proportion of chronic joint-related conditions in the population. This prevalence is higher than the approximately 20% reported arthritis prevalence for the overall U.S. adult population. The difference reflects the composition of the analytic sample rather than a change in national prevalence. Specifically, the analysis was restricted to aged 18–64 years with complete information on all study variables, resulting in a complete-case analytic sample that differs from the overall adult population. Consequently, the observed prevalence should be interpreted as describing the analytic cohort rather than the U.S. adult population. Among key exposures, 21.80% of respondents report vaping, while 65.28% report smoking. Here, we define smoking as those who report having smoked at least 100 cigarettes in their lifetime or currently smokes [61]. Vaping is defined as those use e-cigarettes or other electronic vaping products, categorized as “every day” or “some days,” consistent with standard BRFSS survey coding. The sample is approximately gender-balanced (49.83% male), with 32.97% classified as obese and 66.59% reporting regular exercise. Socioeconomically, a nontrivial share of respondents are in lower-income categories: 35.08% earn less than $25,000 annually, while 38.57% report incomes above $50,000, reflecting notable income heterogeneity. Health comorbidities are also prevalent, including high cholesterol (36.90%), diabetes (13.20%), and asthma (18.66%), all of which are relevant risk factors in chronic disease research. The age distribution skews toward middle-aged and older adults, particularly those aged 50–64 (35.03%), a group typically at higher risk of arthritis and related chronic conditions. Racial composition is predominantly White (79.13%), with smaller shares of Black (6.18%), Hispanic (6.19%), Asian (1.30%), and Indigenous respondents (2.65%). Overall, the descriptive patterns highlight a population with substantial exposure to behavioral and metabolic risk factors, providing a suitable empirical context for examining the relationship between vaping, smoking, and arthritis while accounting for demographic, socioeconomic, and health-related confounders.

**Table 1:** Descriptive statistics.

Variable	Variable Description	Count if 0 (%)	Count if 1 (%)
Arthritis	1 if diagnosed with any form of arthritis and 0 otherwise	23377 (63.58%)	13391 (36.42%)
Vapes	1 if the individual uses any vaping product or e-cigarettes and 0 otherwise	28751 (78.20%)	8017 (21.80%)
Smoke	1 if the individual smokes and 0 otherwise	12765 (34.72%)	24003 (65.28%)
Male	1 if male and 0 otherwise	18446 (50.17%)	18322 (49.83%)
Obesity	1 if obese and 0 otherwise	24646 (67.03%)	12122 (32.97%)
Income less than $25k	1 if has income and less than $25k and 0 otherwise	23869 (64.92%)	12899 (35.08%)
Income between $25k and $35K	1 if Income between $25k and 35K and 0 otherwise	32466 (88.30%)	4302 (11.70%)
Income between $35k and $50K	1 if Income between $35k and $50K and 0 otherwise	31382 (85.35%)	5386 (14.65%)
Income more than 50K	1 if Income above $50K and 0 otherwise	22587 (61.43%)	14181 (38.57%)
Exercise	1 if exercises and 0 otherwise	12286 (33.41%)	24482 (66.59%)
Individual paid any part of the medical cost	1 if the Individual paid any part of the medical cost and 0 otherwise	29655 (80.65%)	7113 (19.35%)
Diabetes	1 if diagnosed with diabetes and 0 otherwise	31916 (86.80%)	4852 (13.20%)
Married	1 if married and 0 otherwise	21751 (59.16%)	15017 (40.84%)
High Cholesterol	1 if diagnosed with high cholesterol and 0 otherwise	23199 (63.10%)	13569 (36.90%)
Age between 18years and 24years	1 if age between 18years and 24years old and 0 otherwise	34900 (94.92%)	1868 (5.08%)
Age between 25years and 29years	1 if age between 25years and 29years old and 0 otherwise	33905 (92.21%)	2863 (7.79%)
Age between 30years and 34years	1 if age between 30years and 34years old and 0 otherwise	33371 (90.76%)	3397 (9.24%)
Age between 35years and 39years	1 if age between 35years and 24years old and 0 otherwise	33122 (90.08%)	3646 (9.92%)
Age between 40years and 49years	1 if age between 40years and 49years old and 0 otherwise	30288 (82.38%)	6480 (17.62%)
Age between 50years and 64years	1 if age between 50years and 64years old and 0 otherwise	23887 (64.97%)	12881 (35.03%)
Cancer	1 if diagnosed with cancer and 0 otherwise	36235 (98.55%)	533 (1.45%)
White	1 if race is white and 0 otherwise	7675 (20.87%)	29093 (79.13%)
Black	1 if race is black and 0 otherwise	34495 (93.82%)	2273 (6.18%)
Asian	1 if race is Asian and 0 otherwise	36291 (98.70%)	477 (1.30%)
Indian	1 if the race is Indian and 0 otherwise	35794 (97.35%)	974 (2.65%)
Hispanic	1 if Hispanic and 0 otherwise	34491 (93.81%)	2277 (6.19%)
Angina or coronary heart disease	1 if diagnosed with Angina or coronary heart disease and 0 otherwise	34432 (93.65%)	2336 (6.35%)
Kidney Disease	1 if diagnosed with kidney disease and 0 otherwise	35351 (96.15%)	1417 (3.85%)
Asthma	1 if diagnosed with asthma and 0 otherwise	29907 (81.34%)	6861 (18.66%)
Alcohol	1 if consumes regular alcohol and 0 otherwise	15003 (40.80%)	21765 (59.20%)

**Note:** Values are reported as counts, with the corresponding percentage for each category presented in parentheses. N = 36768.

## Method

In this study, we examine arthritis as the primary outcome and consider vaping and smoking as the key exposures of interest. Both share the same set of control variables, including year fixed effects for year-specific shocks, thereby allowing a fair comparison. Additionally, the complexity of the BRFSS survey design was accounted for by applying the final sampling weights using a normalized frequency-weighted pseudo-population approach. To account for the complex survey design of the BRFSS, the final sampling weights (_LLCPWT) were incorporated into all analyses following CDC guidelines [60]. As standard machine learning algorithms do not natively support complex survey designs, sampling weights were normalized to sum to the analytic sample size, creating a weighted population that preserves the representativeness of the sample while remaining compatible with the DML estimation framework [62,63]. We first estimate the association of vaping while adjusting for all covariates but excluding smoking, then estimate the association of smoking while adjusting for all covariates but excluding vaping. Next, we estimate the independent association of each behavior by including the other exposure as a control (vaping while controlling for smoking, and smoking while controlling for vaping) along with all other covariates. Finally, we estimate the interactive joint exposure of both vaping and smoking (vaping × smoking) while controlling for both behaviors. This design allows us to examine both joint and independent relationships between each behavior and arthritis.

Estimating these associations is complicated by the risk of model misspecification and high‐dimensional confounding, as many individual‐level factors (e.g., age, health conditions, lifestyle behaviors) may jointly influence exposure status and outcomes via an unknown functional form. Failure to account for these can bias the estimates. To address this challenge, we employ Double Machine Learning (DML) [64], which combines machine learning with orthogonalization to produce consistent estimates even in high-dimensional settings. DML offers advantages over traditional econometric models by avoiding strong parametric assumptions [65], flexibly modeling exposure and outcome relationships, and addressing potential misspecification [66]. Its structure can be combined with various standard supervised ML methods, making it well‐suited for evaluating multiple exposure [64,67] while controlling for a rich set of covariates.

### Double machine learning model

The model is as follows [64]:


$$\begin{array}{c} Y=f_{\text{0}}(X)+\underset{\mu }{\underbrace{D\theta_{\text{0}}+\varepsilon }},\;E\left[\varepsilon |X,\;D\right]=\text{0} \end{array}$$ (1)

$$\begin{array}{c} D=z_{\text{0}}(X)+v,\;E\left[v|X\right]=\text{0} \end{array}$$ (2)

Where Y is the outcome variable, *D* is the exposure variable, which is a dummy variable equal to 1 if exposed and 0 otherwise. *X* is a vector of control variables, $X_{\text{1}},\;\ldots ,\;X_{p}$, while ε and v are error terms. Our parameter of interest is $\theta_{\text{0}}$ which we interpret as conditional association parameter measuring the DML-estimated relationship between exposure and outcome. Correct estimation of $\theta_{\text{0}}$ requires correctly recovering the residuals *ε* and *v*, which in turn requires correctly learning the unknown functions $f_{\text{0}}(X)=E(Y-\;D\theta_{\text{0}}|X)$ and $z_{\text{0}}(X)=E(D|X)$. Because these functions may be nonlinear and high-dimensional, we use machine learning models to discover these two functions— hence the term “Double” Machine Learning. By not imposing parametric assumptions on $f_{\text{0}}$ and $z_{\text{0}}$, we eliminate the possibility of incorrect model specifications and allow for potential nonlinear associations of *X* in the conditional expectation functions [66,68].

However, machine learning models tend to overfit: if the same data used to estimate $f_{\text{0}}(X)$ and $z_{\text{0}}(X)$ are also used to estimate $\theta_{\text{0}}$, the overfitted predictions would push residuals towards zero, absorbing variation that belongs to $D\theta_{\text{0}}$ and biasing the final estimate. To prevent this, we estimate the machine learning models on one part of the data and use those estimates to predict on a separate held-out part, repeating this process across subsamples so that predictions are on average bias-free. This yields correctly recovered residuals and therefore a consistent estimate of $\theta_{\text{0}}$. The technical name for isolating these residuals from the influence of *X* is orthogonalization, and the procedure of learning the functions of *X* in one subsample and predicting in another is called sample splitting with cross-fitting.

To correctly identify the association of interest in the presence of confounders, DML relies on orthogonalization and sample splitting. Specifically, DML randomly divides the full sample into two equal sub-samples, I and $I^{c}$ (each of size $n=\;\frac{N}{\text{2}}$). Using the auxiliary sample $I^{c}$, we first estimate $f_{\text{0}}(X)$ by applying machine learning to predict Y from X alone (excluding *D*). This allows us to construct the outcome residual, $\hat{\mu }=Y-\;\hat{f_{\text{0}}}(X)$, which captures the component $D\theta_{\text{0}}+\;\varepsilon$, i.e., the variation in Y not explained by X, including any effect of D.

Next, still using the auxiliary sample $I^{c}$, we estimate $z_{\text{0}}(X)$ by applying machine learning to predict D from X, and obtain the exposure residual $\hat{v}=D-\hat{z_{\text{0}}}(X)$. Finally, in the primary sample I, we regress $\hat{\mu }$ on $\hat{v}$ using ordinary least squares (OLS) to obtain $\theta_{\text{0}}$:


$$\begin{array}{c} \hat{\mu }=h_{\text{0}}+\hat{v}\theta_{\text{0}}+\gamma \end{array}$$ (3)

This residual-on-residual regression orthogonalizes the influence of X, thereby removing regularization bias and ensuring that prediction errors in machine learning-estimated nuisance components do not contaminate the estimate of $\theta_{\text{0}}$. The estimator of $\theta_{\text{0}}$ is robust and remains consistent even in high-dimensional settings. The estimator $\theta_{\text{0}}$ is consistent if either of the two equations (1) or (2) is estimated correctly [64,67,69]. This double robustness property makes the DML approach more reliable than other methods for cross-sectional analysis [64,66].

To mitigate potential omitted-variable bias, we adjusted for a broad set of demographics, socioeconomic, behavioral, and clinical characteristics, including gender, age, marital status, race, obesity status, income, exercise, out-of-pocket medical expenditures, diabetes, cholesterol level, cancer, heart disease, kidney disease, asthma, and alcohol consumption. These covariates were selected a priori based on prior epidemiological and clinical literature identifying them as established risk factors for arthritis and as recognized correlates of tobacco- and nicotine-use behavior, consistent with covariates adjusted for in prior smoking–arthritis research [21–23,48,52,70]. Although unmeasured factors may still influence the observed relationships, the DML framework flexibly accounts for complex, potentially nonlinear associations among the observed covariates. Accordingly, the reported estimates should be interpreted as conditional associations between exposure and arthritis.

From an implementation standpoint, the choice of machine learning models used in the nuisance equations is critical. In DML, the exposure model (equation 2) is particularly important because it ensures that exposure residuals are orthogonal to X. Errors in outcome prediction (equation 1) primarily affect efficiency, whereas errors in exposure prediction (equation 2) can directly bias the exposure association parameter. Accordingly, we prioritized predictive accuracy in the exposure equation and employed a high-performing model—LightGBM—as the primary learner for predicting the exposure variables (vaping and smoking), based on its superior predictive performance in our validation analysis. For the remaining nuisance components, we combined this approach with penalized regression methods: Lasso regression for continuous residual modeling and Lasso-regularized logistic regression for binary classification tasks. Estimation was carried out using the Partially Linear Regression (PLR) version of DML, and all algorithms were implemented with cross-validation to mitigate overfitting and ensure stable estimates.

To determine whether the adjusted associations of vaping and smoking with arthritis differed, we conducted a Wald test of the null hypothesis that the estimated coefficients for vaping and smoking were equal ($H_{\text{0}}:\;\theta_{vape}=\;\theta_{smoke})$. Two comparisons were performed. First, our primary analysis compared the weighted estimates from the model that included both vaping and smoking, along with the same set of covariates, to estimate the association of each exposure while adjusting for the other. Second, as a supplementary analysis, we compared the weighted estimates from models in which vaping and smoking were each estimated separately without adjustment for the alternate exposure, while adjusting for the same set of covariates. Because the coefficients being compared were estimated from the same study sample and involved overlapping covariate adjustment, they are statistically correlated. The Wald tests accounted for this covariance, whereas comparing point estimates or overlapping confidence intervals alone does not provide a valid statistical comparison. These analyses were conducted to evaluate whether the observed association for vaping differed significantly from that for smoking under both modeling approaches. See S2 File. Wald Test Equation and S3 Fig. Overlapping plots for exposure probabilities under Supporting information for more details.

### Data preprocessing

The data is pre-processed by dropping missing observations, encoding categorical variables into a suitable format, checking correlation among predictors using pairwise correlation matrix, standardizing features, and fixing imbalances using the Synthetic Minority Over-sampling Technique (SMOTE) [71,72]. Missing observations were addressed using complete-case (listwise) deletion, as the DML pipeline does not natively accommodate missing values. Complete-case deletion was preferred over imputation to avoid introducing model-based assumptions into the covariate distribution. A high correlation might generate high variance and overfitting, affecting interpretability. Though our model can handle overfitting, a high correlation might complicate the calculation of variable importance. As shown in Fig 1, the pairwise correlation matrix indicates that absolute correlations among predictors are generally below 0.50.

**Fig 1:**
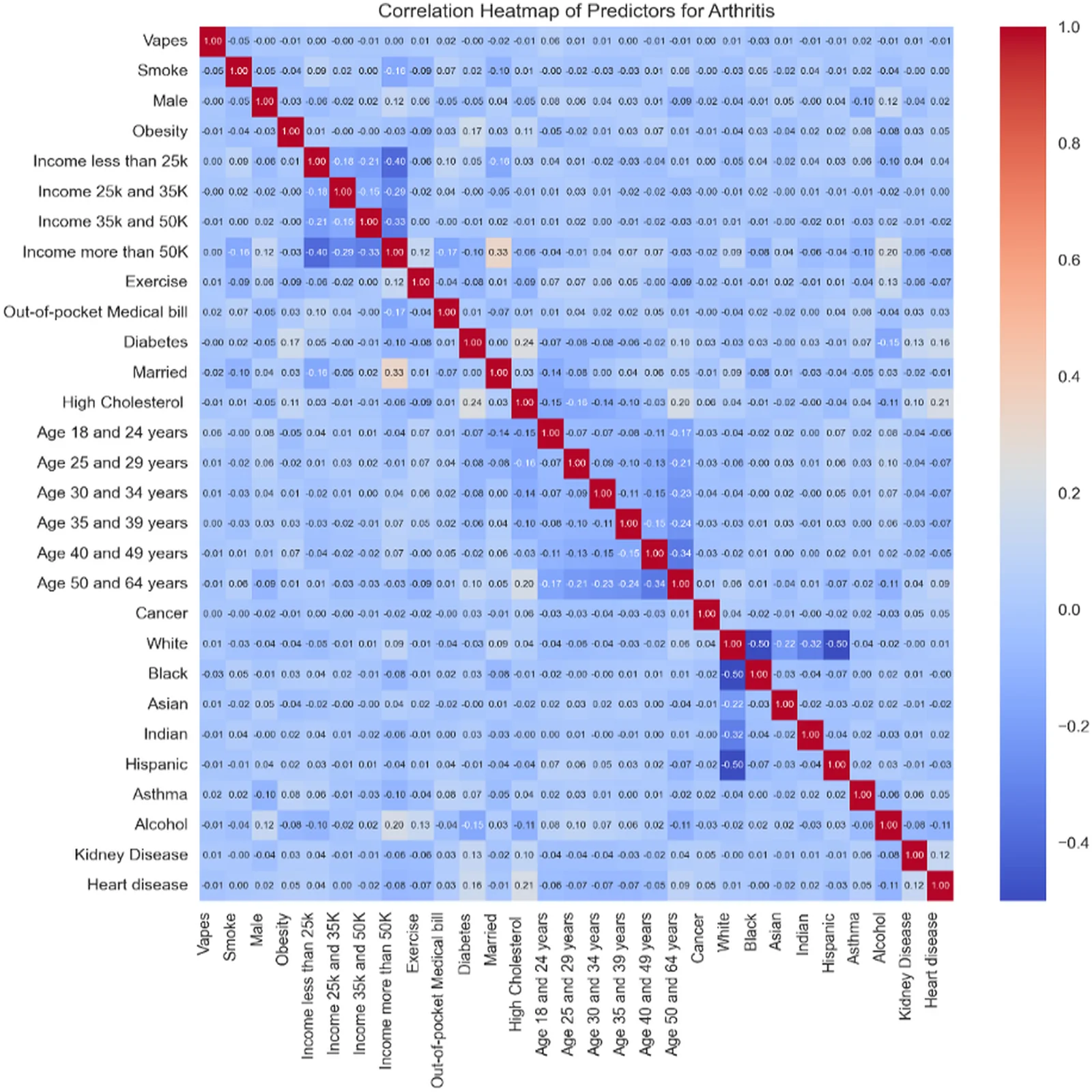
Correlation matrix among predictors. The figure presents a correlation heatmap of the predictor variables used in the arthritis analysis. Each cell shows the pairwise correlation coefficient between variables such as vaping, smoking, demographic characteristics, income categories, health conditions, and behavioral factors. The color scale ranges from blue (negative correlation) to red (positive correlation), with most coefficients clustered near zero, indicating generally weak linear relationships among the predictors. This pattern suggests limited correlation across covariates, with only modest correlations observed within related categories such as income brackets and age groups, which is expected given their mutually exclusive or structured nature.

SMOTE was applied exclusively during the model-selection stage to address class imbalance in the outcome variable when comparing candidate machine-learning models. Specifically, it was used to mitigate the influence of class imbalance on model training and to facilitate a more balanced evaluation of predictive performance [73–76]. Importantly, SMOTE was not applied within the DML estimation pipeline. Because the target association parameter is defined with respect to the observed data-generating process, estimation was conducted using the original sample to preserve the empirical covariate distribution and common-support region, thereby ensuring that the estimated associations were derived from observed rather than synthetically generated covariate patterns. Similarly, the features are standardized for prediction to avoid any possible scale effects. In the ML predict, we randomly split the data into 75% training and 25% testing [77].

Fig 1 shows the pairwise correlation heatmap of all predictors in the arthritis models. The pairwise correlation matrix was computed using Pearson correlation coefficients among all predictors. Pearson’s method was selected because it provides a standardized measure of linear association [74–76]. Overall, the matrix shows low-to-moderate correlations across most covariates, indicating that the predictors provide largely distinct information rather than duplicating the same signal. The most notable patterns occur within mutually exclusive indicator groups, such as income categories, age groups, and race/ethnicity indicators, where negative correlations are expected because membership in one category mechanically reduces the likelihood of membership in another. Importantly, the absence of widespread high correlations (i.e., severe multicollinearity) supports the stability of machine learning estimation and reduces the risk that the model’s predictive performance is driven by redundant features. This diagnostic is especially relevant for SHAP-based interpretation, because when predictors are highly correlated, SHAP values can be harder to interpret, and importance may be “shared” across correlated variables, making it difficult to distinguish each variable’s independent contribution. Thus, the correlation structure shown here strengthens confidence that subsequent ML and SHAP explanations reflect meaningful and separable feature effects, improving the transparency and interpretability of the arthritis prediction results.

### Predicting arthritis with machine learning

A critical component of DML is selecting the best predictive machine learning model to characterize the association between the treatment and other variables. Because the underlying relationships are potentially nonlinear and unknown, we compare multiple models to identify the model that outperforms others in out-of-sample prediction. To ensure a comprehensive search, we first evaluate 15 machine learning models on 75% of the data (training set) using 10–fold cross-validation. The models include LightGBM, XGBoost, Gradient Boosting, Random Forest, Extra Trees, AdaBoost, Logistic Regression, Ridge Classifier, Linear Discriminant Analysis, Quadratic Discriminant Analysis, Naïve Bayes, k-Nearest Neighbors, Decision Tree, Support Vector Machine (linear kernel), and a Dummy Classifier as a baseline. In cross-validation, the training set is partitioned into ten equal subsamples; nine are used for training and one for validation, with the process repeated ten times so that each subsample serves as validation once. Hyperparameters are tuned via grid search, with updates based on the average performance across the ten folds. Complete grid-search results are available upon request. The predictive performance of the 15 models is then evaluated on the remaining 25% of the data (blind test set). The workflow is summarized in Fig 2. To gain further insight into the role of individual predictors, we extract SHAP (Shapley Additive exPlanations) values from the chosen model. Finally, this best-performing model is incorporated into the DML procedure. Additional methodological details are presented as Supporting information (See S1 File).

**Fig 2:**
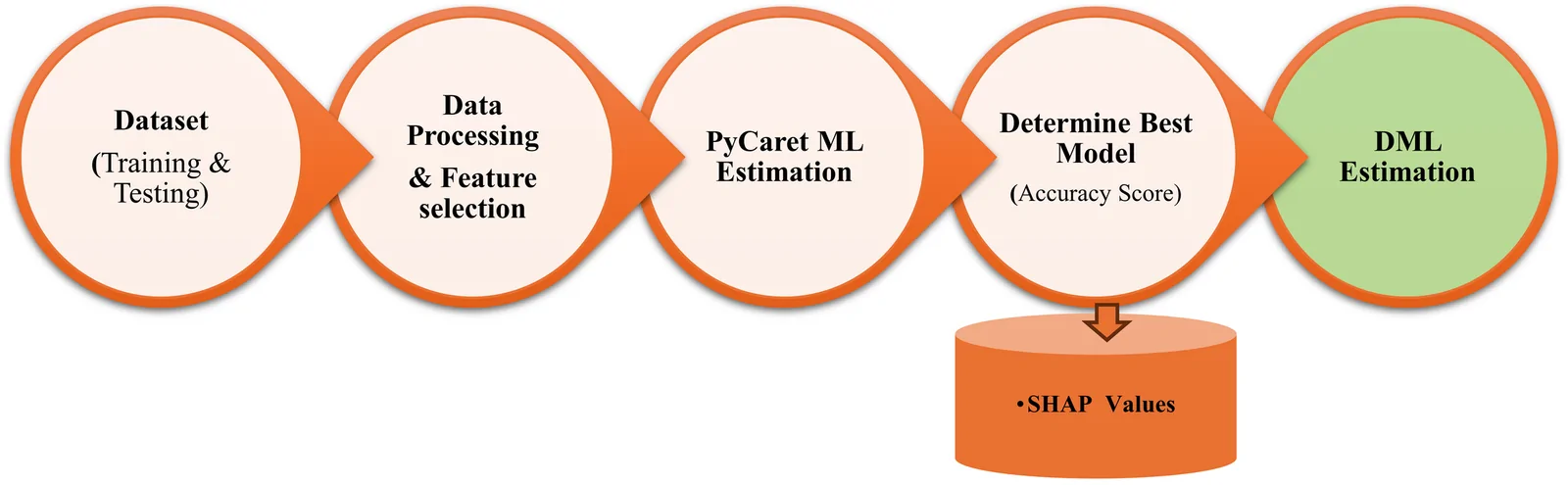
Overall framework. Overview of the analytical workflow, illustrating the sequential pipeline from dataset preparation (training and testing), data preprocessing and feature selection, machine learning model estimation and selection based on predictive performance, SHAP-based interpretability, and final estimation using Double Machine Learning (DML).

These steps interact in a structured sequence designed to separate model selection, nuisance estimation, and association estimation into distinct, non-overlapping stages. Specifically, the train–test split (75% and 25%) and 10-fold cross-validation operate exclusively at the model selection stage, where the best-performing ML model is identified prior to any DML estimation. Once the optimal model is selected, it is passed into the DML procedure, where sample splitting partitions the dataset into auxiliary and primary subsamples (I and $I^{c}$). The auxiliary sample is used solely for nuisance parameter estimation (predicting the outcome and exposure from covariates) while the primary sample is reserved for the residual-on-residual regression that yields the association parameter θ. Cross-fitting then exchanges the roles of both subsamples and aggregates estimates to reduce finite-sample bias. Critically, cross-validation at the model selection stage and DML sample splitting serve distinct purposes: the former optimizes predictive accuracy of the learner, while the latter ensures orthogonalization and prevents overfitting from contaminating the association estimate. Together, these steps form a coherent pipeline in which each component addresses a specific source of bias or model uncertainty.

### Best-performing machine learning model

Among the 15 models evaluated, the Light Gradient Boosting Machine (LightGBM) emerged as the most predictive model. LightGBM is a gradient boosting algorithm that creates a more efficient decision tree algorithm by precomputing histograms of predictor variables or features [78,79]. Its predictive function can be expressed as [80]:


$$\begin{array}{c} {\hat{y}}_{i}^{LG}=\;\sum_{p=\text{1}}^{P}f_{p}\left(x_{i}\right) \end{array}$$ (4)

$f_{p}$ is the tree while p is the number of trees. The aim is to minimize the objective function and get $f_{p}$ which is:


$$\begin{array}{c} f_{p}\;=\text{arg}{min}_{f_{p}\;}\sum_{t=\text{1}}^{n}L\left(y_{i},{\hat{y}}_{i}^{LG(p)}\right)+\phi {(f}_{p}) \end{array}$$ (5)

The function *L* is the loss function, while ϕ is the regularization function given as:


$$\begin{array}{c} \phi {(f}_{p})=\;\alpha T+\frac{\text{1}}{\text{2}}\lambda \sum_{j=\text{1}}^{T}w_{j}^{\text{2}}\; \end{array}$$ (6)

where α and λ are the regularization parameters that penalize leaves T and the weight *w*. The samples are scanned to find the optimal splitting point to calculate the information gain.

The fifteen (15) ML models are estimated concurrently to predict the effect of features on the target variable. The target variable is binary and is evaluated by accuracy. The model with the best accuracy score is selected.

### Contribution of predictors using SHapley Additive exPlanations (SHAP) values

Subsequently, we employ the most predictive model to compute the SHAP values [81], which forecasts the marginal contribution of each variable to our target variable [75]. The mean absolute SHAP values are calculated for each feature, and the value is multiplied by the sign of the correlation coefficient to get the direction of the absolute SHAP value. A calculated positive SHAP value shows that the feature improves the prediction, while a negative value shows that the feature value reduces the forecast.

The SHAP values are determined by aggregating all potential subsets $z^{\prime }$ of the feature set $x^{\prime }$. This comprises the original set of features $x^{\prime }$ and all possible subsets obtained by removing one or more features. Furthermore, *M* is the total number of features. This is estimated using:


$$\begin{array}{c} \emptyset_{i}(f,x)=\;\sum_{z^{\prime }\subseteq x^{\prime }}\frac{\left|z^{\prime }\right|!\left(M-\left|z^{\prime }\right|-\text{1}\right)!}{M!}[f_{x}\left(z^{\prime }\right)-f_{x}\left(z^{\prime }\backslash i\right) \end{array}$$ (8)

Here, $f_{x}\left(z^{\prime }\right)$ is the model output for subset $z^{\prime }$, and $f_{x}\left(z^{\prime }\backslash i\right)$ is the output when feature i is removed. $\left|z^{\prime }\right|$ is the number of features in subset $z^{\prime }$, and the factorial terms weight contributions across all possible feature orderings.

### Illustrative economic scenarios for vaping and smoking association with arthritis

To contextualize the potential economic implications of vaping and smoking associated with arthritis, this study presents illustrative cost projections derived from published unit cost estimates and epidemiological case count ranges. These figures are not intended as precise burden-of-illness estimates; rather, they serve as exploratory scenarios to highlight the order of magnitude of potential costs and to motivate future cost-of-illness research using individual-level data [28].

**Number of Additional Cases** *= Prevalence of Treatment × Population Size ×* θ

**Illustrative Cost Projection** *= Number of Additional Cases × Average Cost per outcome per person*

It is important to emphasize that this analysis is strictly illustrative and does not establish causality; rather, it translates the observed conditional associations between tobacco-related exposures and arthritis into approximate population-level and economic estimates to contextualize their potential public health significance. The first step estimates the number of arthritis cases statistically associated with vaping and smoking in the United States based on the observed associations. The total potential cost association is then quantified by monetizing these estimated health associations. Given the observational nature of the data, these figures should be interpreted as illustrative upper-bound approximations and are subject to the limitations of residual confounding and non-random exposure assignment inherent to cross-sectional designs.

To strengthen the robustness of the projected economic burden estimates and account for uncertainty in cost parameters, we conducted deterministic sensitivity analyses using conservative annual arthritis cost estimates derived from prior U.S.-based studies [82–85]. Cost inputs were selected from nationally representative, peer-reviewed sources and inflation-adjusted to comparable U.S. dollar values where applicable [82–84]. The analysis incorporated the full range of arthritis-related expenditures, spanning conservative direct medical costs to upper-bound societal cost estimates. Our primary analysis emphasized the conservative lower-bound scenario, which captures only verified direct healthcare expenditures while excluding indirect productivity losses, disability-related costs, and broader societal costs. This approach provides a deliberately conservative and methodologically reproducible benchmark for population-level cost projection. Anchoring the analysis to this lower-bound estimate minimizes the risk of overestimation and enhances the credibility and policy relevance of the findings.

Data on the U.S. population aged 18–64 are sourced from the U.S. Census Bureau [86]. The prevalence of vaping is estimated to range between 4.5% and 6.0% [12,14,85], while current smoking prevalence is reported to be between 11.7% and 15.5% [87]. Additionally, the study incorporates estimates of the average annual cost per arthritis patient of $700 for conservative, base and upper bound cost respectively [82,83,88,89].

### Placebo test estimation

To further ascertain consistency in our estimates, we conducted a placebo DML test. Here, we created a placebo treatment by drawing a random sample from our data using a Bernoulli distribution where *p* is the mean of the treatment and *n* is the number of observations in each model. For example, when vaping is our treatment variable, we used *p* = 0.218 and *n* = 36,768. The main goal of the placebo DML test is to check whether the observed associated treatment effects in our analysis are likely due to genuine underlying relationships rather than spurious correlations or overfitting.

## Results

The machine learning (ML) prediction algorithm is estimated using 15 models commonly applied in the economics literature, with the best-performing model for each prediction selected for optimization based on the accuracy score (See S4 Table in the Supporting information for the 15 ML estimation results). LightGBM emerges as the best model for predicting arthritis, achieving approximately 75.1% accuracy. The model is subsequently tuned and optimized using 10-fold cross-validation and hyperparameter adjustment. Hyperparameters are tuned via grid search, with updates based on average performance across the ten folds (see S5 Table in the Supporting information for the 10-fold cross-validation results). The optimized LightGBM model improved in performance with an increased accuracy of approximately 76.3% (See S6 Table in the Supporting information).

### SHAP values

Fig 3 shows that obesity, out-of-pocket medical bills, and exercise are the top predictors of the prevalence of arthritis in our model, with obesity and out-of-pocket medical bills contributing positively to arthritis complications while exercise contributes negatively to arthritis. Additionally, income level is heterogeneously associated with the prevalence of arthritis; income above $50,000 per year is negatively associated, while below $50,000 annual income is positively associated. The marginal contributions of the remaining variables in the prediction task are low.

**Fig 3:**
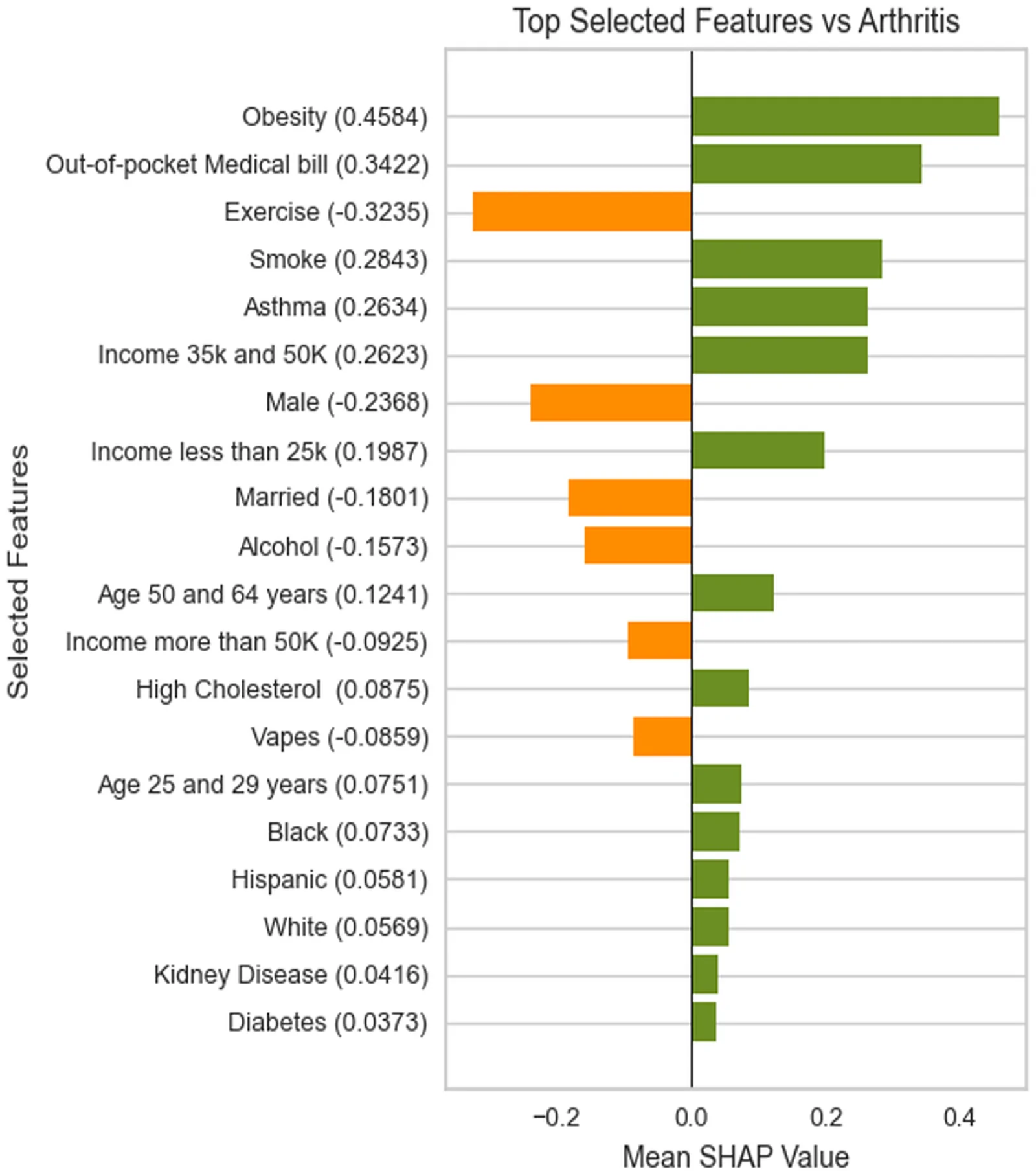
SHAP values for arthritis. This shows the mean absolute SHAP values for the top-selected features, with the correlation signs indicated. The figure shows each feature’s non-conditional marginal contribution to the prediction outcome. The horizontal axes show the average absolute SHAP values multiplied by the correlation sign, and the bars show the strength of the association between the predictor and the response variable. The green color represents a positive contribution, while the orange color shows a negative contribution.

### DML result

DML is employed to estimate the conditional association between each exposure variable (vaping and smoking) and the outcome variable (arthritis). In this study, the resulting estimates, while derived using a framework conventionally applied to treatment effect estimation, are strictly interpreted as conditional associations between the exposure variables and arthritis after accounting for the set of observed covariates, and do not imply causality given the cross-sectional and observational nature of the data. The results are presented in Table 2.

**Table 2:** DML results.

DML Estimate (*Ɵ*)					
Dependent Variable	*Exposure = Vape* *(No Smoke Control)*	*Exposure = Vape* *(With Smoke Control)*	*Exposure = Smoke* *(No Vape Control)*	*Exposure = Smoke* *(With Vape Control)*	*Exposure = Vape* × *Smoke* *(Vape and Smoke Control)*
Arthritis	0.0191*** (0.0021)	0.0212*** (0.0025)	0.0214*** (0.0022)	0.0222*** (0.0022)	0.0233*** (0.00104)
**Unweighted Exposure (*Ɵ*)**					
Arthritis	0.0276*** (0.0048)	0.0277*** (0.0054)	0.0113*** (0.0048)	0.0131*** (0.0048)	0.0308*** (0.0066)

*** p < 0.01, ** p < 0.05, * p < 0.1

Standard error in parenthesis

The weighted estimates show that individuals who vape have an arthritis prevalence that is approximately 1.91 percentage points higher than that of individuals who do not vape. When smoking behavior is included as an additional control variable, the association between vaping and arthritis remains positive, with an estimated 2.12 percentage point higher prevalence among vapers relative to non-vapers. Similarly, individuals classified as severe or current smokers are associated with an arthritis prevalence that is approximately 2.22 percentage points higher than that of individuals who are not current or severe smokers when vaping is included as a control variable. When vaping is not included as a control variable, the estimated association for smoking is slightly attenuated to 2.14 percentage points. Individuals who both vape and smoke are associated with an arthritis prevalence that is approximately 2.33 percentage points higher than that of individuals who do not engage in dual use, holding other observed factors constant.

A Wald test was conducted to compare the weighted estimated associations for vaping and smoking in the model that simultaneously adjusted each exposure for the other. The difference between the two estimated associations was not statistically significant (z = −0.98, p = 0.328). A supplementary Wald test comparing the weighted estimates from models that did not adjust for the alternate exposure likewise found no statistically significant difference (z = −0.65, p = 0.516). Together, these findings indicate that the observed association between vaping and arthritis did not differ significantly from that observed for smoking under either model specification.

As a robustness analysis, Table 2 also reports unweighted association estimates to assess the robustness of the findings to the survey weighting procedure. The unweighted estimates are largely consistent with the weighted estimates in terms of direction and statistical significance, with all associations remaining positive and significant at the 1% level. The unweighted association between vaping and arthritis is estimated at 2.76 percentage points without smoking as a control and 2.77 percentage points with smoking as a control, compared to 1.91 and 2.12 percentage points in the weighted specifications, respectively. For smoking, the unweighted estimates are slightly attenuated relative to the weighted estimates, at 1.13 percentage points without vaping as a control and 1.31 percentage points with vaping as a control, compared to 2.14 and 2.22 percentage points in the weighted specifications. The unweighted joint exposure estimate is 3.08 percentage points, compared to 2.33 percentage points in the weighted specification. The observed differences in magnitude between weighted and unweighted estimates are consistent with the expected impact of survey weight normalization, which corrects for the overrepresentation of certain demographic subgroups in the raw BRFSS sample. Importantly, the directional consistency and sustained statistical significance across both specifications confirm that the primary findings are robust to the weighting procedure and are not an artifact of the survey design.

It is important to note that the directional sign displayed in Fig 3 should not be interpreted as the adjusted association estimated by the DML model. The SHAP figure reports mean absolute feature contributions whose displayed direction is derived from the raw correlation between each predictor and its SHAP values within the prediction model. By contrast, the DML estimates quantify the conditional association between vaping and arthritis after adjustment for smoking and the full set of observed covariates. Consequently, the negative directional sign for vaping in Fig 3 reflects the unadjusted predictive relationship within the machine-learning model rather than the adjusted association reported by DML. Differences in sign are therefore expected when confounding variables, particularly age, smoking behavior, and comorbidities, alter the conditional association after adjustment.

### Illustrative economic scenarios for vaping and smoking association with arthritis

Using the weighted estimates, we illustrate the potential economic significance of the observed associations between tobacco-related exposures and arthritis, with emphasis on the conservative lower-bound direct medical cost scenario of $700 per person. Midpoint estimates are presented alongside uncertainty ranges derived from lower- and upper-bound case count estimates; these represent scenario-based uncertainty bounds rather than statistically derived confidence intervals, consistent with the illustrative nature of these projections.

For arthritis cases statistically associated with vaping, ranging from approximately 190,581–254,109 (midpoint: 222,345), the conservative illustrative potential cost projection is estimated at approximately $155.6 million ($133.4 million–$177.9 million). For arthritis cases statistically associated with smoking, ranging from approximately 518,886–687,413 (midpoint: 603,150), the conservative illustrative potential cost projection is estimated at approximately $422.2 million ($363.2 million–$481.2 million). When considered jointly, the combined conservative illustrative potential cost projection for arthritis cases statistically associated with vaping and smoking is estimated at approximately $577.8 million ($496.6 million–$659.1 million) annually. Uncertainty ranges were consistent across both exposure groups, spanning 27.9% to 28.6% of their respective midpoint estimates, confirming that the width of these projections is driven by case-count variability rather than cost assumptions, which were held constant within the conservative scenario. Base-case and upper-bound scenario results are reported in full in S7 Table of the Supporting information.

It must be emphasized that these figures do not represent causal estimates of the economic cost of vaping and smoking on arthritis; rather, they provide an illustrative quantification of the potential economic significance of the observed associations, under the assumption that those associations reflect a meaningful relationship between these exposures and arthritis outcomes. For context, the total cost of arthritis in the United States has been reported to range between $303.5 billion and $550.2 billion, with affected individuals losing an estimated 13–20 workdays per year [90,91]. Under the conservative cost framework, the projected economic burden statistically attributable to vaping and smoking corresponds to approximately 0.09% to 0.22% of total arthritis-related expenditure. Although modest under direct healthcare cost assumptions alone, these estimates represent a meaningful and potentially avoidable component of the national arthritis burden. When broader indirect and societal costs are incorporated, the projected economic burden attributable to vaping and smoking increases substantially, accounting for up to 9.23% of total arthritis-related costs. These findings underscore the potentially disproportionate downstream economic implications of tobacco and nicotine exposure beyond immediate clinical outcomes. This finding underscores the potential financial and public health significance of the observed associations between vaping, smoking, and arthritis. Policymakers should find these estimates informative when designing targeted interventions aimed at reducing tobacco-related exposures and mitigating associated healthcare costs, while acknowledging the inherent uncertainty of association-based economic projections.

### Placebo test result

By generating a placebo treatment variable that follows the distribution of the treatment variable but is unrelated to the outcome, we can verify from Table 3 that the DML procedure does not systematically produce significant effects in the absence of a true associated treatment effect.

**Table 3:** Placebo results.

Dependent Variable	DML Estimate (θ)			
	*Exposure = Vape* *(No Smoke Control)*	*Exposure = Vape* *(With Smoke Control)*	*Exposure = Smoke* *(No Vape Control)*	*Exposure = Smoke* *(With Vape AControl)*
Arthritis	−0.0042 (0.0054)	−0.0048 (0.0054)	0.0076 (0.0047)	0.0071 (0.0047)

*** p < 0.01, ** p < 0.05, * p < 0.1

Standard error in parenthesis

## Discussion and conclusion

This study employed DML to estimate the associated treatment effect of vaping and smoking on arthritis. This study reveals that vaping and smoking are associated with a higher prevalence of arthritis and associated with an increased health risk and economic scenarios projecting the potential cost implications. The result further showed that vaping and smoking place a projecting financial weight on the economy at large.

A general finding of this research demonstrates that the act of vaping and smoking are independently associated with arthritis. The presence of harmful substances in both vaping and smoking appears to be associated with the aggravation of the pain and suffering felt by those with arthritis, thereby contributing to arthritis issues throughout the body.

A unique and important finding of this study is the robustness of the DML estimates across alternative specifications that account for concurrent nicotine behaviors. Specifically, the estimated association of vaping on arthritis remains positive and largely unchanged when smoking is included as a control, and the smoking effect exhibits a similarly stable magnitude after controlling for vaping. This stability indicates that the observed associations are not driven by dual-use confounding but instead reflect independent and additive relationships between each behavior and arthritis. Although smoking shows a marginally larger associated size than vaping, the close magnitude of the estimates suggests that vaping is not a risk-neutral alternative with respect to arthritis risk. From a public health perspective, this finding is particularly noteworthy given widespread misperceptions about the safety of e-cigarettes, which may encourage sustained or unrestricted use under the assumption of reduced harm. Such behavioral patterns, combined with evidence that vaping can facilitate progression to combustible cigarette use, may link to cumulative health risks and long-term disease problem [47,92].

Another important finding is that concurrent vaping and smoking is associated with higher arthritis prevalence (0.0233 percentage points relative to adults who are not dual users), suggesting that vaping does not eliminate the health risks associated with combustible tobacco use. Because this estimate uses all non-dual users as the reference group, it is not directly comparable with the single-exposure estimates, and we do not draw conclusions about whether the dual-use association is greater or smaller than the sum of the individual associations.

Beyond the direct health implication, the findings of this study highlight a substantial associated potential cost implications of arthritis associated with vaping and smoking. The findings further emphasize that both vaping and smoking play a critical role in impairing arthritis-related issues, underscoring the urgent need for awareness, preventive measures, and policy interventions to mitigate their associated influences.

Our findings broadly align with the existing literature documenting a positive association between smoking and arthritis [21–23,48,70], while extending this evidence by showing that vaping is also significantly associateAd with higher arthritis prevalence as well as a significant joint exposure. The estimated associations in our study are meaningful in magnitude and consistent with prior observational research reporting 1.4 to 2.4 percentage point increases in arthritis-related complications among smokers [52,70]. Similarly, a recent study has associated vaping to a 1.31 to 2.06 percentage point increase in inflammatory arthritis complications [29]. The consistency of these estimates across model specifications, including mutual adjustment for smoking and vaping, further strengthens the robustness of the findings and indicates that the observed relationships are not solely driven by behavioral overlap or model sensitivity.

Methodologically, this study contributes uniquely to literature by employing a DML framework on a large, nationally representative dataset to examine the independent and joint associations of smoking and vaping with arthritis. Prior comparative evidence has largely relied on systematic meta-analyses [93–95], trend analyses [59–61], simulation models [47], and qualitative assessments [25], which often face limitations in addressing high-dimensional confounding and complex behavioral interdependence. By contrast, the DML approach allows for flexible control of a rich set of socioeconomic, demographic, and health-related covariates while reducing sensitivity to functional form misspecification and omitted variable bias. This is particularly important in the context of smoking and vaping, as both behaviors are jointly determined by multiple correlated factors that are also associated with chronic health outcomes. To our knowledge, this is among the first studies to jointly assess the differential and joint associations of vaping and smoking with arthritis using a data-driven, high-dimensional framework.

This study faces a few limitations that should be noted. First, the analysis is based on cross-sectional observational data; therefore, the findings should be interpreted as associations rather than causal effects. As with all cross-sectional studies, residual confounding, reverse causality, and the absence of temporal ordering between exposures and outcomes cannot be ruled out.Individuals experiencing chronic arthritis pain or functional limitations may initiate or maintain smoking or vaping as a coping mechanism, leading to an observed association even if tobacco use did not precede disease onset. The present data cannot distinguish this pathway from the hypothesized direction in which vaping or smoking precedes arthritis. Prospective longitudinal studies are therefore needed to establish temporal ordering and better evaluate the direction of the observed associations. Second, despite that BRFSS provides extensive information on demographic, socioeconomic, behavioral, and health characteristics, some potentially important confounders are unavailable. These include occupational physical demands, prior joint injury, dietary quality, and opioid or pain medication use, all of which may be associated with both tobacco-related exposures and arthritis outcomes. Several of these omitted factors would be expected to strengthen the observed associations, thereby introducing upward bias. At the same time, because the analysis is conducted at the population level, idiosyncratic individual-level sources of unobserved heterogeneity may partially offset one another if their effects are approximately mean-zero. Nevertheless, to the extent that the omitted factors identified above are systematically related to both tobacco use and arthritis, the net bias is more likely to be upward than downward. Consequently, the reported estimates may be interpreted as conservative upper-end approximations of the underlying population associations. In addition, sensitivity analyses based on separate current- and former-smoker classifications could not be conducted because the analytic BRFSS variables used in this study does not have detailed smoking-history information. Also, all measures, including arthritis status and tobacco-related behaviors, are self-reported and may therefore be subject to recall error, reporting bias, or misclassification. In addition, the analysis was restricted to adults aged 18–64 years to ensure sufficient variation in vaping and smoking behaviors and to maintain a more behaviorally comparable sample. Consequently, the findings are most directly applicable to the working-age, noninstitutionalized adult population and may not generalize to younger or older age groups. Plus, because the BRFSS samples the civilian, noninstitutionalized population, individuals residing in institutional settings, such as nursing homes and long-term care facilities, are excluded. Finally, although survey weights were incorporated using a normalized population approach consistent with CDC guidance, the DML framework does not natively accommodate design-based variance estimation using primary sampling units and strata. These considerations may affect the external validity and precision of the reported estimates.

In conclusion, the observed association between vaping and arthritis did not differ significantly from that observed for smoking. These findings do not support the assumption that vaping is associated with a lower prevalence of arthritis than smoking. The results contribute to the growing evidence on the public health implications of vaping and inform ongoing discussions regarding its role as an alternative to combustible cigarettes. We recommend that future studies employ longitudinal data to validate and compare with our estimates.

## Supporting information

S1 File Machine learning model. (PDF)

S2 File Wald test equation. (PDF)

S3 Fig Overlapping plot. (PDF)

S4 Table 15 ML prediction for arthritis. (PDF)

S5 Table Arthritis: 10–fold cross validation. (PDF)

S6 Table Optimized model evaluation metrics. (PDF)

S7 Table Illustrative economic scenarios. (PDF)

## References

[1] Yanbaeva DG, et al. Systemic effects of smoking. Chest. 2007;131(5):1557–66. 10.1378/chest.06-2179 17494805

[2] Gonçalves RB, Coletta RD, Silvério KG, Benevides L, Casati MZ, da Silva JS, et al. Impact of smoking on inflammation: Overview of molecular mechanisms. Inflamm Res. 2011;60(5):409–24. doi: 10.1007/s00011-011-0308-7 21298317

[3] West R. Tobacco smoking: Health impact, prevalence, correlates and interventions. Psychol Health. 2017;32(8):1018–36. doi: 10.1080/08870446.2017.1325890 28553727 PMC5490618

[4] Schuller HM. Impact of neuro-psychological factors on smoking-associated lung cancer. Cancers (Basel). 2014;6(1):580–94. doi: 10.3390/cancers6010580 24633083 PMC3980616

[5] Kondo T, Nakano Y, Adachi S, Murohara T. Effects of tobacco smoking on cardiovascular disease. Circ J. 2019;83(10):1980–5. doi: 10.1253/circj.CJ-19-0323 31462607

[6] Duncan MS, Freiberg MS, Greevy RA Jr, Kundu S, Vasan RS, Tindle HA. Association of smoking cessation with subsequent risk of cardiovascular disease. JAMA. 2019;322(7):642–50. doi: 10.1001/jama.2019.10298 31429895 PMC6704757

[7] Mambo A, Yang Y, Mahulu E, Zihua Z. Investigating the interplay of smoking, cardiovascular risk factors, and overall cardiovascular disease risk: NHANES analysis 2011-2018. BMC Cardiovasc Disord. 2024;24(1):193. doi: 10.1186/s12872-024-03838-7 38575889 PMC10993506

[8] Jeong S-M, Yoo JE, Park J, Jung W, Lee KN, Han K, et al. Smoking behavior change and risk of cardiovascular disease incidence and mortality in patients with type 2 diabetes mellitus. Cardiovasc Diabetol. 2023;22(1):193. doi: 10.1186/s12933-023-01930-4 37516874 PMC10387213

[9] Banks E, Joshy G, Korda RJ, Stavreski B, Soga K, Egger S, et al. Tobacco smoking and risk of 36 cardiovascular disease subtypes: fatal and non-fatal outcomes in a large prospective Australian study. BMC Med. 2019;17(1):128. doi: 10.1186/s12916-019-1351-4 31266500 PMC6607519

[10] Tyas SL, Koval JJ, Pederson LL. Does an interaction between smoking and drinking influence the risk of Alzheimer’s disease? Results from three Canadian data sets. Stat Med. 2000;19(11–12):1685–96. doi: 10.1002/(sici)1097-0258(20000615/30)19:11/12<1685::aid-sim454>3.0.co;2-# 10844727

[11] Centers for Disease Control and Prevention. Smoking & tobacco use: Fast facts and fact sheets. 2024. https://www.cdc.gov/tobacco/data_statistics/fact_sheets/index.htm

[12] Vahratian A, et al. Electronic cigarette use among adults in the United States, 2019-2023. NCHS Data Brief. 2025;524. 10.15620/cdc/174583 PMC12035660 40036117

[13] CDC. E-Cigarette Use Among Youth. 2024.

[14] Kramarow EA, Elgaddal N. Current electronic cigarette use among adults aged 18 and over: United States, 2021. 2023. 37486729

[15] Rhoades DA, Comiford AL, Dvorak JD, Ding K, Hopkins M, Spicer P, et al. Vaping patterns, nicotine dependence and reasons for vaping among American Indian dual users of cigarettes and electronic cigarettes. BMC Public Health. 2019;19(1):1211. doi: 10.1186/s12889-019-7523-5 31477072 PMC6721166

[16] Yong H, Borland R, Cummings KM, Gravely S, Thrasher JF, McNeill A, et al. Reasons for regular vaping and for its discontinuation among smokers and recent ex‐smokers: findings from the 2016 ITC Four Country Smoking and Vaping Survey. Addiction. 2019;114(S1):35–48. doi: 10.1111/add.14593 PMC6717696 30821861

[17] NHS. Vaping to quit smoking - Better Health. 2024.

[18] CDC. Electronic cigarettes (E-cigarettes). 2023. https://www.cdc.gov/tobacco/basic_information/e-cigarettes.html

[19] Rajbhandari-Thapa J, Chike OE, Lyford C. Economic aspects of public health nutrition across the life cycle. Life cycle nutrition for public health professionals. 2024. p. 367.

[20] CDC. Arthritis. Centers for Disease Control and Prevention. 2023.

[21] Saag KG, Cerhan JR, Kolluri S, Ohashi K, Hunninghake GW, Schwartz DA. Cigarette smoking and rheumatoid arthritis severity. Ann Rheum Dis. 1997;56(8):463–9. doi: 10.1136/ard.56.8.463 9306868 PMC1752420

[22] Chang K, Yang SM, Kim SH, Han KH, Park SJ, Shin JI. Smoking and rheumatoid arthritis. Int J Mol Sci. 2014;15(12):22279–95. doi: 10.3390/ijms151222279 25479074 PMC4284707

[23] Vittecoq O, et al. The impact of smoking on rheumatoid arthritis outcomes. 2018;135–8. 10.1016/j.jbspin.2017.12.004 29246527

[24] Becker TD, Arnold MK, Ro V, Martin L, Rice TR. Systematic review of electronic cigarette use (vaping) and mental health comorbidity among adolescents and young adults. Nicotine Tob Res. 2021;23(3):415–25. doi: 10.1093/ntr/ntaa171 32905589

[25] Blank M-L, Hoek J. Smoking, vaping and drinking: A qualitative analysis of Aotearoa New Zealand young adults who tried e-cigarettes to stop smoking tobacco. Addiction. 2024;119(4):686–95. doi: 10.1111/add.16413 38114132

[26] Cheng K-W, Shang C, Lee HM, Chaloupka FJ, Fong GT, Borland R, et al. Costs of vaping: evidence from ITC four country smoking and vaping survey. Tob Control. 2021;30(1):94–7. doi: 10.1136/tobaccocontrol-2019-055344 32086385 PMC7507861

[27] Jonas A. Impact of vaping on respiratory health. BMJ. 2022;378. 10.1136/bmj-2021-065997 35851281

[28] Tatangelo M, Tomlinson G, Paterson JM, Keystone E, Bansback N, Bombardier C. Health care costs of rheumatoid arthritis: A longitudinal population study. PLoS One. 2021;16(5):e0251334. doi: 10.1371/journal.pone.0251334 33956894 PMC8101709

[29] Tian Y, Jiao Z, Mao Y, Zhang Z. E-cigarette usage and arthritis in the United States, a nationwide cross-sectional survey. Front Pharmacol. 2022;13:883550. doi: 10.3389/fphar.2022.883550 35685635 PMC9170919

[30] Nicholson T, Scott A, Newton Ede M, Jones SW. Do E-cigarettes and vaping have a lower risk of osteoporosis, nonunion, and infection than tobacco smoking?. Bone Joint Res. 2021;10(3):188–91. doi: 10.1302/2046-3758.103.BJR-2020-0327.R1 33709767 PMC7998067

[31] Peruzzi M, Biondi-Zoccai G, Carnevale R, Cavarretta E, Frati G, Versaci F. Vaping Cardiovascular Health Risks: an Updated Umbrella Review. Curr Emerg Hosp Med Rep. 2020;8(3):103–9. doi: 10.1007/s40138-020-00219-0 32837803 PMC7296287

[32] Fryman C, Lou B, Weber AG, Steinberg HN, Khanijo S, Iakovou A, et al. Acute respiratory failure associated with vaping. Chest. 2020;157(3):e63–8. doi: 10.1016/j.chest.2019.10.057 32145818

[33] Braymiller JL, Barrington-Trimis JL, Leventhal AM, Islam T, Kechter A, Krueger EA, et al. Assessment of nicotine and cannabis vaping and respiratory symptoms in young adults. JAMA Netw Open. 2020;3(12):e2030189. doi: 10.1001/jamanetworkopen.2020.30189 33351085 PMC7756238

[34] Viswam D, Trotter S, Burge PS, Walters GI. Respiratory failure caused by lipoid pneumonia from vaping e-cigarettes. BMJ Case Rep. 2018;2018:bcr2018224350. doi: 10.1136/bcr-2018-224350 29982176 PMC6040540

[35] Cherian C, Buta E, Simon P, Gueorguieva R, Krishnan-Sarin S. Association of vaping and respiratory health among youth in the Population Assessment of Tobacco and Health (PATH) study wave 3. Int J Environ Res Public Health. 2021;18(15):8208. doi: 10.3390/ijerph18158208 34360499 PMC8346152

[36] Shahandeh N, Chowdhary H, Middlekauff HR. Vaping and cardiac disease. Heart. 2021;107(19):1530–5. doi: 10.1136/heartjnl-2020-318150 33574049

[37] Tsai M, Byun MK, Shin J, Crotty Alexander LE. Effects of e-cigarettes and vaping devices on cardiac and pulmonary physiology. J Physiol. 2020;598(22):5039–62. doi: 10.1113/JP279754 32975834 PMC10506663

[38] Kuntic M, et al. Could e-cigarette vaping contribute to heart disease? Expert Rev Respir Med. 2020;14(11):1131–9. 10.1080/17476348.2020.1807332 32757856

[39] Cooper MT, Pesko MF. The effect of e-cigarette indoor vaping restrictions on adult prenatal smoking and birth outcomes. J Health Econ. 2017;56:178–90. doi: 10.1016/j.jhealeco.2017.10.002 29107198 PMC5733637

[40] Shi H, Fan X, Horton A, Haller ST, Kennedy DJ, Schiefer IT, et al. The effect of electronic-cigarette vaping on cardiac function and angiogenesis in mice. Sci Rep. 2019;9(1):4085. doi: 10.1038/s41598-019-40847-5 30858470 PMC6411855

[41] Figueredo CA, Abdelhay N, Figueredo CM, Catunda R, Gibson MP. The impact of vaping on periodontitis: A systematic review. Clin Exp Dent Res. 2021;7(3):376–84. doi: 10.1002/cre2.360 33274850 PMC8204026

[42] Yang JJ, Piper ME, Indic P, Buu A. Statistical methods for predicting e-cigarette use events based on beat-to-beat interval (BBI) data collected from wearable devices. Stat Med. 2024;43(17):3227–38. doi: 10.1002/sim.10124 38816901 PMC11250691

[43] Wadsworth E, Neale J, McNeill A, Hitchman SC. How and why do smokers start using e-cigarettes? qualitative study of vapers in London, UK. Int J Environ Res Public Health. 2016;13(7):661. doi: 10.3390/ijerph13070661 27376312 PMC4962202

[44] Al-Hamdani M, Manly E. Smoking cessation or initiation: The paradox of vaping. Prev Med Rep. 2021;22:101363. doi: 10.1016/j.pmedr.2021.101363 33868902 PMC8044675

[45] McGrath-Morrow SA, et al. The effects of nicotine on development. Pediatrics. 2020;145(3). 10.1542/peds.2019-1346 PMC7049940 32047098

[46] American Lung Association. Nicotine. 2023.

[47] Levy DT, Tam J, Sanchez-Romero LM, Li Y, Yuan Z, Jeon J, et al. Public health implications of vaping in the USA: the smoking and vaping simulation model. Popul Health Metr. 2021;19(1):19. doi: 10.1186/s12963-021-00250-7 33865410 PMC8052705

[48] Baka Z, Buzás E, Nagy G. Rheumatoid arthritis and smoking: putting the pieces together. Arthritis Res Ther. 2009;11(4):238. doi: 10.1186/ar2751 19678909 PMC2745780

[49] Chen N, Fong DYT, Wong JYH. Health and economic burden of low back pain and rheumatoid arthritis attributable to smoking in 192 countries and territories in 2019. Addiction. 2024;119(4):677–85. doi: 10.1111/add.16404 38105035

[50] Gerli R, Sherer Y, Vaudo G, Schillaci G, Gilburd B, Giordano A, et al. Early atherosclerosis in rheumatoid arthritis: effects of smoking on thickness of the carotid artery intima media. Ann N Y Acad Sci. 2005;1051:281–90. doi: 10.1196/annals.1361.069 16126969

[51] Salis Z. Investigation of the associations of smoking with hip osteoarthritis: a baseline cross-sectional and four- to five-year longitudinal multicohort study. ACR Open Rheumatol. 2024;6(3):155–66. doi: 10.1002/acr2.11644 38174808 PMC10933634

[52] Kong L, Wang L, Meng F, Cao J, Shen Y. Association between smoking and risk of knee osteoarthritis: a systematic review and meta-analysis. Osteoarthritis Cartilage. 2017;25(6):809–16. doi: 10.1016/j.joca.2016.12.020 28011100

[53] Zhu S. Association of smoking and osteoarthritis in US (NHANES 1999–2018). Sci Rep. 2023;13(1):3911. 10.1038/s41598-023-30644-6 PMC9995311 36890196

[54] Ni J, Wang P, Yin K-J, Huang J-X, Tian T, Cen H, et al. Does smoking protect against developing osteoarthritis? Evidence from a genetically informed perspective. Semin Arthritis Rheum. 2022;55:152013. doi: 10.1016/j.semarthrit.2022.152013 35500427

[55] Gheisary Z. Association of sleep quality and general, mental, and oral health with lifestyle traits (dietary intake, smoking status) in arthritis: A cross-sectional study from the Canadian Community Health Survey (CCHS). Nutrients. 2024;16(13):2091. 10.3390/nu16132091 PMC11243648 38999838

[56] Pennington N. Psoriatic arthritis: A case study. The Journal for Nurse Practitioners. 2024;20(6):105014.

[57] Daas C. The effects of e-vapor on the pathogenesis of rheumatoid arthritis mouse models and potential implications for future generations. 2020.

[58] Nicholson T, Scott A, Newton Ede M, Jones SW. The impact of E-cigarette vaping and vapour constituents on bone health. J Inflamm (Lond). 2021;18(1):16. doi: 10.1186/s12950-021-00283-7 33952248 PMC8097983

[59] Fiani B, Noblett C, Nanney JM, Gautam N, Pennington E, Doan T, et al. The impact of “vaping” electronic cigarettes on spine health. Cureus. 2020;12(6):e8907. doi: 10.7759/cureus.8907 32742873 PMC7389958

[60] CDC. Behavioral Risk Factor Surveillance System Survey Data. 2022.

[61] National Center for Health Statistics. National Health Interview Survey. 2017.

[62] Lumley T. Complex surveys: a guide to analysis using R. John Wiley & Sons. 2011.

[63] Ridgeway G, Kovalchik SA, Griffin BA, Kabeto MU. Propensity score analysis with survey weighted data. J Causal Inference. 2015;3(2):237–49. doi: 10.1515/jci-2014-0039 29430383 PMC5802372

[64] Chernozhukov V, et al. Double/debiased machine learning for treatment and structural parameters. The Econometrics Journal. 2018;:C1–68.

[65] Lechner M, Mareckova J. Comprehensive Causal Machine Learning. arXiv preprint. 2024. doi: arXiv:2405.10198

[66] Jung Y, Tian J, Bareinboim E. Estimating identifiable causal effects through double machine learning. AAAI. 2021;35(13):12113–22. doi: 10.1609/aaai.v35i13.17438

[67] Knaus MC. Double machine learning-based programme evaluation under unconfoundedness. The Econometrics Journal. 2022;25(3):602–27. doi: 10.1093/ectj/utac015

[68] Chernozhukov V, et al. Double/debiased machine learning for treatment and structural parameters. Oxford, UK: Oxford University Press. 2018.

[69] Fuhr J, Berens P, Papies D. Estimating causal effects with double machine learning--a method evaluation. arXiv preprint. 2024. doi: arxiv:2403.14385

[70] Alfredsson L, Klareskog L, Hedström AK. Influence of smoking on disease activity and quality of life in patients with rheumatoid arthritis: results from a Swedish case–control study with longitudinal follow‐up. Arthritis Care & Research. 2023;75(6):1269–77. 10.1002/acr.25026 36149365

[71] Sowjanya AM, Mrudula O. Effective treatment of imbalanced datasets in health care using modified SMOTE coupled with stacked deep learning algorithms. Appl Nanosci. 2023;13(3):1829–40. doi: 10.1007/s13204-021-02063-4 35132368 PMC8811587

[72] Wang K, Tian J, Zheng C, Yang H, Ren J, Li C, et al. Improving risk identification of adverse outcomes in chronic heart failure using SMOTE+ENN and machine learning. Risk Manag Healthc Policy. 2021;14:2453–63. doi: 10.2147/RMHP.S310295 34149290 PMC8206455

[73] He HB, et al. ADASYN: Adaptive Synthetic Sampling Approach for Imbalanced Learning. In: 2008 IEEE International Joint Conference on Neural Networks, 2008. 1322–8.

[74] Amin MD, Badruddoza S, McCluskey JJ. Predicting access to healthful food retailers with machine learning. Food Policy. 2021;99:101985. doi: 10.1016/j.foodpol.2020.101985 33082618 PMC7564312

[75] Oduniyi OS, Chike OE, Antwi MA. Machine learning and econometric analysis of the heterogeneous welfare effects of agricultural cooperative membership in Mpumalanga, South Africa. Scientific African. 2026;31:e03230. doi: 10.1016/j.sciaf.2026.e03230

[76] Chike OE, Oduniyi OS, Nwaiwu JI. An interpretable machine learning approach to predicting depression and diabetes. Healthcare Analytics. 2026;9:100454. doi: 10.1016/j.health.2026.100454

[77] Sindhwani N, Rana A, Chaudhary A. Breast cancer detection using machine learning algorithms. In: 2021 9th International conference on reliability, Infocom technologies and optimization (trends and future directions) (ICRITO). IEEE; 2021.

[78] Ke G, et al. Lightgbm: A highly efficient gradient boosting decision tree. Advances in neural information processing systems. 2017;30.

[79] Fan J, Ma X, Wu L, Zhang F, Yu X, Zeng W. Light Gradient Boosting Machine: An efficient soft computing model for estimating daily reference evapotranspiration with local and external meteorological data. Agricultural Water Management. 2019;225:105758. doi: 10.1016/j.agwat.2019.105758

[80] Sibindi R, Mwangi RW, Waititu AG. A boosting ensemble learning based hybrid light gradient boosting machine and extreme gradient boosting model for predicting house prices. Engineering Reports. 2023;5(4):e12599.

[81] Shapley LS. 17. A value for n-person games. Contributions to the Theory of Games (AM-28), Volume II. Princeton University Press. 1953. p. 307–18. doi: 10.1515/9781400881970-018

[82] Bitton R. The economic burden of osteoarthritis. Am J Manage Care. 2009;15(8 Suppl):S230-5. 19817509

[83] Lo J, Chan L, Flynn S. A systematic review of the incidence, prevalence, costs, and activity and work limitations of amputation, osteoarthritis, rheumatoid arthritis, back pain, multiple sclerosis, spinal cord injury, stroke, and traumatic brain injury in the United States: A 2019 Update. Arch Phys Med Rehabil. 2021;102(1):115–31. doi: 10.1016/j.apmr.2020.04.001 32339483 PMC8529643

[84] Hresko A, Lin T-C, Solomon DH. Medical care costs associated with rheumatoid arthritis in the US: A systematic literature review and meta-analysis. Arthritis Care Res (Hoboken). 2018;70(10):1431–8. doi: 10.1002/acr.23512 29316377 PMC6033682

[85] Obisesan OH, et al. Trends in e-cigarette use in adults in the United States, 2016-2018. JAMA Internal Medicine. 2020;180(10):1394–8. 10.1001/jamainternmed.2020.2817 PMC7489391 32897288

[86] Census Bureau US. United States - Census Bureau: Quick Facts. 2025. https://data.census.gov/profile/United_States?g=010XX00US Accessed 2025 March 24.

[87] American Lung Association. Tobacco trends brief: Overall tobacco trends brief. 2025. https://www.lung.org/research/trends-in-lung-disease/tobacco-trends-brief/overall-smoking-trends

[88] Lo TKT, Parkinson L, Cunich M, Byles J. Cost of arthritis: a systematic review of methodologies used for direct costs. Expert Rev Pharmacoecon Outcomes Res. 2016;16(1):51–65. doi: 10.1586/14737167.2016.1126513 26618446

[89] Leifer VP, Katz JN, Losina E. The burden of OA-health services and economics. Osteoarthritis Cartilage. 2022;30(1):10–6. doi: 10.1016/j.joca.2021.05.007 34023527 PMC8605034

[90] Gunnarsson C, Chen J, Rizzo JA, Ladapo JA, Naim A, Lofland JH. The employee absenteeism costs of rheumatoid arthritis: evidence from US National Survey Data. J Occup Environ Med. 2015;57(6):635–42. doi: 10.1097/JOM.0000000000000461 26053366

[91] Loud EE, Duong HT, Henderson KC, Reynolds RM, Ashley DL, Thrasher JF, et al. Addicted to smoking or addicted to nicotine? A focus group study on perceptions of nicotine and addiction among US adult current smokers, former smokers, non-smokers and dual users of cigarettes and e-cigarettes. Addiction. 2022;117(2):472–81. doi: 10.1111/add.15634 34227709 PMC8733050

[92] Levy DT, Warner KE, Cummings KM, Hammond D, Kuo C, Fong GT, et al. Examining the relationship of vaping to smoking initiation among US youth and young adults: a reality check. Tob Control. 2019;28(6):629–35. doi: 10.1136/tobaccocontrol-2018-054446 30459182 PMC6860409

[93] Hassanein ZM, Barker AB, Murray RL, Britton J, Agrawal S, Leonardi-Bee J. Impact of smoking and vaping in films on smoking and vaping uptake in adolescents: systematic review and meta-analysis. Health Educ Behav. 2022;49(6):1004–13. doi: 10.1177/10901981221086944 35502561 PMC9578073

[94] Ruffin L, Swilley BR, Berg JA, Douglass B, Ruffin W, Padden-Denmead M. The chronic nature of smoking and vaping: a comprehensive analysis. J Nurse Pract. 2024;20(2):104903. doi: 10.1016/j.nurpra.2023.104903

[95] Rehan HS, Maini J, Hungin AP. Vaping versus smoking: a quest for efficacy and safety of e-cigarette. Current Drug Safety. 2018;13(2):92–101. 10.2174/1574886313666180227110556 29485005

