# Supplementary material for: Vaping and smoking as risk factors for arthritis: Evidence from double machine learning
Source: PLoS One. 2026 Oct 23;21(10):e0360863. doi: 10.1371/journal.pone.0360863 (PMC13693875; doi:10.1371/journal.pone.0360863)
Supplement: Supplementary file 1 [file pone.0360863.s001.pdf]

S1. Machine Learning Model

We compared 15 machine learning algorithms that differ in how they learn patterns from data. The model with the highest accuracy score is optimized using 10-fold cross-validation and hyperparameters. Among tree-based ensemble models, Light Gradient Boosting Machine (LightGBM) achieved the highest accuracy (75%) and AUC (85%). LightGBM builds decision trees sequentially, using histogram-based feature binning to speed up computation while reducing memory cost, making it especially efficient on large datasets. XGBoost and Gradient Boosting follow a similar boosting approach, where each new tree focuses on correcting the errors of prior trees, but are slightly less efficient computationally. Random Forest and Extra Trees instead rely on bagging: they grow many trees in parallel on bootstrapped samples of the data and aggregate their predictions. While robust and less prone to overfitting than single decision trees, they generally performed weaker than boosting algorithms. AdaBoost also builds trees sequentially, but reweights misclassified observations at each step, which helps with difficult cases; however, it was not as accurate as gradient boosting methods in this context.

Linear and regression-based models provided more interpretability but lagged behind ensembles. Ridge Classifier, Logistic Regression, and Linear Discriminant Analysis (LDA) all attempt to fit linear decision boundaries, with Ridge applying L2 regularization to avoid overfitting. Their performance hovered around 70% accuracy with moderate AUC. Quadratic Discriminant Analysis (QDA) relaxes LDA's linearity assumption, allowing quadratic boundaries, and Naïve Bayes relies on probabilistic rules under independence assumptions. Both methods captured high recall (over 81% in QDA's case) meaning they identified arthritis cases well, but did so at the expense of precision, misclassifying many non-arthritis cases.

Other algorithms, such as k-Nearest Neighbors (KNN), classify new cases based on the majority class among the closest training samples, which can adapt to nonlinear boundaries but is computationally heavier and sensitive to noisy data. Decision Trees alone produced the least stable results among tree-based methods, often overfitting to the training sample. Support Vector Machines (SVM) with a linear kernel work by finding the hyperplane that best separates the two groups; they achieved strong recall but lower precision, reflecting difficulty balancing errors across groups. Finally, the Dummy Classifier, which predicts classes by random guessing or simple rules like class proportions, served as a baseline and showed no meaningful predictive power.

Overall, boosting algorithms, particularly LightGBM, consistently outperformed classical linear and probabilistic approaches by offering better accuracy, AUC, and balance across precision and recall. This confirms that flexible, tree-based ensembles are better suited for predicting arthritis in our dataset than traditional statistical models.
