# Supplementary material for: Vaping and smoking as risk factors for arthritis: Evidence from double machine learning
Source: PLoS One. 2026 Oct 23;21(10):e0360863. doi: 10.1371/journal.pone.0360863 (PMC13693875; doi:10.1371/journal.pone.0360863)
Supplement: Supplementary file 2 [file pone.0360863.s002.pdf]

S2. Wald Test Equation

$$z = \frac{\hat{\theta}_{vape} - \hat{\theta}_{smoke}}{\sqrt{\widehat{Var}(\hat{\theta}_{vape}) + \widehat{Var}(\hat{\theta}_{smoke}) - 2\widehat{Cov}(\hat{\theta}_{vape}, \hat{\theta}_{smoke})}},$$

Where $\widehat{Var}(\hat{\theta}_{vape})$ and $\widehat{Var}(\hat{\theta}_{smoke})$ are the estimated variances of the two DML coefficients, and $\widehat{Cov}(\hat{\theta}_{vape}, \hat{\theta}_{smoke})$ is their covariance, obtained from the sample covariance of the paired observation-level influence functions $\psi_{vape,i}$ and $\psi_{smoke,i}$:

$$\widehat{Cov}(\hat{\theta}_{vape}, \hat{\theta}_{smoke}) = \frac{1}{n} \widehat{Cov}(\psi_{vape,i}, \psi_{smoke,i})$$

Under $H_0: \theta_{vape} = \theta_{smoke}$, z is asymptotically standard normal.
