# Supplementary material for: Vaping and smoking as risk factors for arthritis: Evidence from double machine learning
Source: PLoS One. 2026 Oct 23;21(10):e0360863. doi: 10.1371/journal.pone.0360863 (PMC13693875; doi:10.1371/journal.pone.0360863)
Supplement: Supplementary file 3 [file pone.0360863.s003.pdf]

**S5. Table. Arthritis: 10–Fold Cross Validation**

| Fold | Accuracy | AUC | Recall | Prec. | F1 | Kappa | MCC |
|---|---|---|---|---|---|---|---|
| 0 | 0.7461 | 0.8357 | 0.7403 | 0.7488 | 0.7446 | 0.4922 | 0.4922 |
| 1 | 0.7450 | 0.8451 | 0.7350 | 0.7498 | 0.7423 | 0.4899 | 0.4900 |
| 2 | 0.7457 | 0.8409 | 0.7304 | 0.7533 | 0.7417 | 0.4915 | 0.4917 |
| 3 | 0.7438 | 0.8405 | 0.7307 | 0.7506 | 0.7405 | 0.4877 | 0.4878 |
| 4 | 0.7537 | 0.8415 | 0.7443 | 0.7587 | 0.7514 | 0.5074 | 0.5075 |
| 5 | 0.7552 | 0.8461 | 0.7367 | 0.7652 | 0.7507 | 0.5104 | 0.5108 |
| 6 | 0.7365 | 0.8379 | 0.7130 | 0.7482 | 0.7302 | 0.4730 | 0.4736 |
| 7 | 0.7506 | 0.8383 | 0.7304 | 0.7611 | 0.7454 | 0.5011 | 0.5015 |
| 8 | 0.7456 | 0.8404 | 0.7365 | 0.7502 | 0.7433 | 0.4913 | 0.4913 |
| 9 | 0.7612 | 0.8512 | 0.7380 | 0.7739 | 0.7555 | 0.5224 | 0.5230 |
| **Mean** | 0.7483 | 0.8418 | 0.7335 | 0.7560 | 0.7446 | 0.4967 | 0.4970 |
| **Std** | 0.0066 | 0.0043 | 0.0081 | 0.0081 | 0.0067 | 0.0132 | 0.0133 |
