# Supplementary material for: Vaping and smoking as risk factors for arthritis: Evidence from double machine learning
Source: PLoS One. 2026 Oct 23;21(10):e0360863. doi: 10.1371/journal.pone.0360863 (PMC13693875; doi:10.1371/journal.pone.0360863)
Supplement: Supplementary file 4 [file pone.0360863.s004.pdf]

S4. Table. 15 ML Prediction for Arthritis

S/NO	Model ID	Model	Accuracy	AUC	Recall	Prec.	F1	Kappa	MCC	TT (Sec)
1	lightgbm	Light Gradient Boosting Machine	0.7507	0.8455	0.7349	0.7589	0.7467	0.5014	0.5017	0.2450
2	xgboost	Extreme Gradient Boosting	0.7480	0.8412	0.7209	0.7622	0.7410	0.4959	0.4967	0.1730
3	gbc	Gradient Boosting Classifier	0.7405	0.8319	0.7527	0.7348	0.7436	0.4810	0.4811	0.3630
4	rf	Random Forest Classifier	0.7327	0.8101	0.7164	0.7406	0.7283	0.4654	0.4657	0.2840
5	et	Extra Trees Classifier	0.7310	0.7857	0.7001	0.7463	0.7224	0.4620	0.4630	0.4320
6	ada	Ada Boost Classifier	0.7263	0.8112	0.7357	0.7222	0.7288	0.4527	0.4528	0.1270
7	ridge	Ridge Classifier	0.7046	0.7727	0.7486	0.6881	0.7171	0.4092	0.4109	0.0200
8	lda	Linear Discriminant Analysis	0.7046	0.7727	0.7486	0.6881	0.7171	0.4092	0.4109	0.0270
9	lr	Logistic Regression	0.7045	0.7731	0.7399	0.6910	0.7146	0.4089	0.4100	0.0280
10	knn	K Neighbors Classifier	0.6993	0.7616	0.7377	0.6852	0.7105	0.3987	0.3999	0.1710
11	dt	Decision Tree Classifier	0.6983	0.7233	0.6476	0.7209	0.6822	0.3966	0.3987	0.0310
12	svm	SVM - Linear Kernel	0.6904	0.7566	0.7576	0.6680	0.7097	0.3809	0.3848	0.0630
13	qda	Quadratic Discriminant Analysis	0.6845	0.7502	0.8168	0.6459	0.7214	0.3690	0.3827	0.0250
14	nb	Naive Bayes	0.6837	0.7476	0.8052	0.6478	0.7180	0.3673	0.3787	0.0200
15	dummy	Dummy Classifier	0.4999	0.5000	0.3000	0.1499	0.1999	0.0000	0.0000	0.0200
