# Supplementary material for: Vaping and smoking as risk factors for arthritis: Evidence from double machine learning
Source: PLoS One. 2026 Oct 23;21(10):e0360863. doi: 10.1371/journal.pone.0360863 (PMC13693875; doi:10.1371/journal.pone.0360863)
Supplement: Supplementary file 5 [file pone.0360863.s005.pdf]

S5. Table. Arthritis: 10–Fold Cross Validation

Fold	Accuracy	AUC	Recall	Prec.	F1	Kappa	MCC
0	0.7461	0.8357	0.7403	0.7488	0.7446	0.4922	0.4922
1	0.7450	0.8451	0.7350	0.7498	0.7423	0.4899	0.4900
2	0.7457	0.8409	0.7304	0.7533	0.7417	0.4915	0.4917
3	0.7438	0.8405	0.7307	0.7506	0.7405	0.4877	0.4878
4	0.7537	0.8415	0.7443	0.7587	0.7514	0.5074	0.5075
5	0.7552	0.8461	0.7367	0.7652	0.7507	0.5104	0.5108
6	0.7365	0.8379	0.7130	0.7482	0.7302	0.4730	0.4736
7	0.7506	0.8383	0.7304	0.7611	0.7454	0.5011	0.5015
8	0.7456	0.8404	0.7365	0.7502	0.7433	0.4913	0.4913
9	0.7612	0.8512	0.7380	0.7739	0.7555	0.5224	0.5230
**Mean**	0.7483	0.8418	0.7335	0.7560	0.7446	0.4967	0.4970
**Std**	0.0066	0.0043	0.0081	0.0081	0.0067	0.0132	0.0133
