# Supplementary material for: Vaping and smoking as risk factors for arthritis: Evidence from double machine learning
Source: PLoS One. 2026 Oct 23;21(10):e0360863. doi: 10.1371/journal.pone.0360863 (PMC13693875; doi:10.1371/journal.pone.0360863)
Supplement: Supplementary file 6 [file pone.0360863.s006.pdf]

S6. Table. Optimized Model Evaluation Metrics

Dependent Variable	Best Model	Accuracy	AUC	Sensitivity	Precision	F1	Kappa	MCC
Arthritis	LightGBM	0.7626	0.8602	0.7427	0.7735	0.7578	0.5252	0.5256
