# Supplementary material for: Vaping and smoking as risk factors for arthritis: Evidence from double machine learning
Source: PLoS One. 2026 Oct 23;21(10):e0360863. doi: 10.1371/journal.pone.0360863 (PMC13693875; doi:10.1371/journal.pone.0360863)
Supplement: Supplementary file 7 [file pone.0360863.s007.pdf]

## S7. Table. Illustrative Economic Scenarios

| Population | **Conservative Scenario** Direct medical costs only · $700/person | **Base-Case Scenario** Direct + moderate indirect costs · $12,500/person | **Upper-Bound Scenario** Broad societal costs · $29,742/person | **Uncertainty Range** (Full range % of midpoint) |
|---|---|---|---|---|
| **Vaping-associated arthritis cases** | | | | |
| Lower bound (190,581 cases) | **$133.4 million** ($133.4M – $133.4M) | **$2.38 billion** ($2.38B – $2.38B) | **$5.67 billion** ($5.67B – $5.67B) | |
| Midpoint estimate (222,345 cases) | **$155.6 million** ($133.4M – $177.9M) | **$2.78 billion** ($2.38B – $3.18B) | **$6.61 billion** ($5.67B – $7.56B) | **28.6%** |
| Upper bound (254,109 cases) | **$177.9 million** ($177.9M – $177.9M) | **$3.18 billion** ($3.18B – $3.18B) | **$7.56 billion** ($7.56B – $7.56B) | |
| **Smoking-associated arthritis cases** | | | | |
| Lower bound (518,886 cases) | **$363.2 million** ($363.2M – $363.2M) | **$6.49 billion** ($6.49B – $6.49B) | **$15.43 billion** ($15.43B – $15.43B) | |
| Midpoint estimate (603,150 cases) | **$422.2 million** ($363.2M – $481.2M) | **$7.54 billion** ($6.49B – $8.59B) | **$17.94 billion** ($15.43B – $20.45B) | **27.9%** |
| Upper bound (687,413 cases) | **$481.2 million** ($481.2M – $481.2M) | **$8.59 billion** ($8.59B – $8.59B) | **$20.45 billion** ($20.45B – $20.45B) | |
| **Combined vaping and smoking burden** | | | | |
| Lower bound (709,467 cases) | **$496.6 million** ($496.6M – $496.6M) | **$8.87 billion** ($8.87B – $8.87B) | **$21.10 billion** ($21.10B – $21.10B) | |
| Midpoint estimate (825,494 cases) | **$577.8 million** ($496.6M – $659.1M) | **$10.32 billion** ($8.87B – $11.77B) | **$24.55 billion** ($21.10B – $28.00B) | **28.1%** |
| Upper bound (941,522 cases) | **$659.1 million** ($659.1M – $659.1M) | **$11.77 billion** ($11.77B – $11.77B) | **$28.00 billion** ($28.00B – $28.00B) | |

*M = million; B = billion. Full range % of midpoint = (upper bound − lower bound) ÷ midpoint × 100. The ± value represents the half-range deviation from the midpoint. Ranges reflect variability in case-count estimates only; per-person cost inputs are held constant within each scenario column. These figures do not represent causal estimates. They provide an illustrative quantification of the potential economic significance of observed statistical associations between tobacco-related exposures and arthritis under three cost assumptions. Note: These economic scenarios are derived by applying the estimated conditional associations to population prevalence and published cost estimates. They are intended solely to illustrate the potential economic magnitude associated with the observed relationships and should not be interpreted as estimates of costs causally attributable to vaping or smoking.*
